# COVID-19 lockdown implementation in Ghana: lessons learned and hurdles to overcome

**DOI:** 10.1057/s41271-021-00330-w

**Published:** 2022-01-04

**Authors:** Abraham Assan, Hawawu Hussein, David N. K. Agyeman-Duah

**Affiliations:** 1 Global Policy and Advocacy Network (GLOOPLAN), Accra, Ghana; 2 Ho Teaching Hospital, Ho, Ghana; 3 Academic City College, Adabraka, P. O. Box AD 421, Accra, Ghana

**Keywords:** COVID-19, Lockdown implementation, Ghana

## Abstract

COVID-19 exacts huge health and economic burdens on the global economy. To minimize spread of the virus, most governments of the wealthiest countries implemented lockdowns—a tough preventive measure. Ghana implemented a partial lockdown of two major cities, then lifted it in few weeks despite rising numbers of cases. This Viewpoint presents perspectives of key stakeholders in the public about lockdown implementation in Ghana. Respondents characterize the lifting of the lockdown as hasty, poorly communicated, and lacking transparency. Most would have preferred a longer lockdown despite the pressures it imposed especially on the urban poor. Participants expressed uncertainty about the health systems' ability to respond to increases in disease transmission and to provide education, engagement, and empowerment needed in communities, but even so would have preferred a longer lockdown. We offer lessons for more effective policy and implementation of lockdowns.

## Key message

1. What works well in one country may not elsewhere. Governments should implement lockdowns in accord with the WHO criteria for when and how to lift lockdown restrictions.
2. Increasing systems awareness among district managers could contribute to more appropriate, effective, and sustainable solutions.
3. Information on disease outbreaks should be clearly and regularly communicated to all stakeholders to foster an effective and coordinated strategy.
4. Policy makers should also promote community-based interventions to improve population adherence with preventive measures, even in the absence of government-mandated restrictions.

## Introduction

The novel SARS-CoV-2 (COVID-19) is a global public health burden, having caused over 259 million confirmed cases and about 5 million confirmed deaths worldwide as of November 2021. It is the worst pandemic in the century [1]. Experts’ predictions and lay persons’ speculations painted a gloomy picture, claiming the burden would be higher in Africa and might exacerbate conditions of weak health systems on the continent. But the burden across Africa has been lower and less severe than predicted [2]. Some scholars have speculated causes to include the largely young population of Africa, some hidden or cross immunity from other diseases, and warm temperatures [3]. All these factors may have benefitted Ghanaians, and contributed to a relatively lower burden of the disease in the region.

Many countries have instituted restrictive measures on free movement of persons and goods to help limit the spread of the virus. These restrictions created chaos early in the pandemic, forcing people to adjust to a ‘new normal’ and to adhere to the WHO. The Government of Ghana implemented a partial lockdown on March 30, 2020 in its two most populous cities, Accra and Kumasi. The President of the country, His Excellency Nana Addo Dankwa Akufo-Addo, announced the policy and security agencies enforced it. A stay-at-home order applied to almost all people in the two cities and most activities. Exceptions included individuals providing or accessing food, water, beverages, public toilets and baths, health services, electricity, banking, and those in key institutions, such as the media, Members of the Executive, the Legislature, and the Judiciary. The Government lifted the lockdown during the third week of its implementation as numbers of new cases of COVID-19 continued to rise [4]. The abrupt change of policy prompted debates among the public and policy-makers as to whether the lockdown had achieved its intended purpose.

Although researchers have conducted several studies on lockdown globally, none explored the perception of the general public on lockdown implementation in Ghana. This Viewpoint presents perspectives of the public on the lifting of the lockdown and its policy implications in Ghana. We also provide policy recommendations for effective lockdown implementation and preparedness for future outbreaks.

## Key informant interviews

We gathered data from April 23, 2020 to July 1, 2020, immediately after the government lifted the lockdown. We selected a total of 101 key informants through a purposive and snowball sampling approach. We sought interviewees knowledgeable about the policy, the social context, and technical information about the COVID-19 pandemic. Participants included educated community opinion leaders, community members, media personalities, health professionals, academics, and students. Restrictions, such as social or physical distancing, made it infeasible for researchers to conduct face-to-face interviews. We designed interview guides using google forms and administered the survey online, using WhatsApp. We conducted the study in accord with the ethical principles of the Declaration of Helsinki, World Medical Association [5] because the COVID-19 restrictions made it difficult to obtain ethical clearance from the local ethics committee. Participation of this study was entirely voluntary—we informed participants of their right to participate, decline, or withdraw from the study at any time without consequence. Because the study aimed to explore only opinions of participants without exciting strong feelings or emotions, there was no risk for participants. Researchers guaranteed privacy, confidentiality, and anonymity of the information shared by interviewees. Researchers explained the objective of the study and encouraged all participants to read and understand the purpose of the study before consenting to participate. We used a conventional content analysis approach to interpret responses [6], through the lens of the WHO criteria for lifting lockdown. According to the WHO [7], any country considering a lift of lockdown should ensure that:

- Disease transmission is under control.
- Health system is able to “detect, test, isolate, and treat every case and trace every contact.”
- Hotspot risks are minimized in vulnerable places.
- Schools, workplaces, and other essential places have established preventative measures.
- The risk of importing new cases can be managed.
- Communities are fully educated, engaged, and empowered to live under a new normal.

We also explored and analyzed perspectives of stakeholders on factors relevant to the Ghanaian context. We included issues to develop and expand our understanding about the phenomenon within the local context. These pertain to whether:

- The lockdown implementation was appropriate and achieved its objective (contributed to a reduction in the spread of the disease) before it was lifted, or it was lifted prematurely.
- The decision to lift the lockdown was transparent and evidence-driven.
- The decision to lift the lockdown was to achieve political gains or not.
- The health system of Ghana would be able to detect, test, isolate, and treat every case and trace every contact should cases and mortality increase.

## Characteristics of participants and participants’ views

About 57.5% of the respondents were between 33 and 67 years with the mean age of 31.4 ± 8.9, about 58.6% of informants were males, and 57.3% lived in cities that experienced the lockdown. Approximately 10.8% of participants were COVID-19 frontline workers, 49.5% had university degree, 29% had postgraduate education, and 39.1% were senior-level professionals (Table 1).

**Table 1:** Demographic features of research participants

Variables	Freq (%), *N* = 101
Age in years (mean ± SD)	31.4 ± 8.9
18–32	46 (46.5)
33–67	53 (53.5)
Gender	
Male	58 (58.6)
Female	42 (42.4)
Highest education obtained	
Certificate/secondary	6 (6.3)
Diploma	13 (13.7)
Degree	47 (49.5)
Postgraduate	29 (30.5)
Frontline workers	
Yes	10 (10.8)
No	83 (89.2)
Level of professional experience	
Junior	20 (23.0)
Intermediate	33 (37.9)
Senior	34 (39.1)
Location of residence	
Urban settings under lockdown	51 (57.3)
Urban setting not under lockdown	23 (24.5)
Rural settings (were not under lockdown)	20 (21.3)

Most participants (58.2%) agreed with the statement that the government’s decision to lift the lockdown was premature. Most respondents (54.1%) reported agreed that lifting the lockdown was inappropriate because it had not yet achieved its objective of slowing the rate of transmission of the virus. An overwhelming number of respondents (80.6%) asserted that the lifting of lockdown was dangerous and could lead to an escalation in COVID-19 spread. Many interviewees (43.3%) opined that the decision-making processes used in lifting the lockdown lacked transparency. Many (36.7%) believed the decision to lift lockdown was not evidence-driven, about (35.7%) of study participants believed that the decision was evidence-based (Table 2).

**Table 2:** Perception of key informants about the lifting of lockdown in Ghana

Perspective of stakeholders	Total response (*N* = 101 (%))		
	Agree	Disagree	Uncertain
**Questions based on the WHO criteria to lift lockdown**			
Disease transmission is under control?	26 (26.5)	26 (26.5)	48 (46.9)
(Ghana) health system is (currently) able to “detect, test, isolate, and treat every case and trace every contact”	40 (40.8)	30 (30.6)	28 (28.6)
Hotspot risks are minimized in vulnerable places?	31 (32.0)	34 (35.1)	32 (32.0)
Schools, workplaces, and other essential places have established preventative measures	37 (38.1)	26 (26.8)	44 (35.1)
The risk of importing new cases can be managed	60 (60.8)	20 (20.6)	18 (18.6)
Communities are fully educated, engaged, and empowered to live under a new normal	27 (27.8)	24 (24.7)	47 (47.4)
**Context-specific related questions**			
The lifting of the lockdown is appropriate because it has achieved its objective	26 (26.5)	53 (54.1)	19 (19.4)
There is transparency in the methods used in lifting the lockdown	31 (32.0)	42 (43.3)	24 (24.7)
The lifting of lockdown was premature	58 (58.2)	28 (28.6)	12 (12.2)
The lifting of the lockdown is dangerous and could lead to a rise in numbers of cases	79 (80.6)	12 (12.2)	7 (7.1)
Decision to lift lockdown was evidence-driven	35 (35.7)	36 (36.7)	27 (27.6)
Decision to lift lockdown was to achieve political gains	28 (28.6)	45 (45.9)	25 (25.5)
Should COVID-19 cases escalate, the health system of Ghana would be able to detect, test, isolate, and treat every case and trace every contact	13 (22.8)	22 (38.6)	22 (38.6)

Respondents did not believe a desire to achieve political gain motivated the swift decision by government to lift the lockdown. Instead, they reported key reasons to be exacerbation of socioeconomic inequalities of the urban poor and the economic impact of lockdown on the informal sector, the largest source of employment in the country.

The populations of Accra and Kumasi constitute about 17% of the Ghanaian population [8]. In both cities, low-income residents of urban slums comprise a substantial population of artisan workers paid daily wages. Given the important economic and social role residents of Accra and Kumasi play in the civic life of Ghana, participants stressed that the lockdown indirectly had an impact on the entire Ghanaian economy.

Some interviewees (40.8%) emphasized the ability of the health system of Ghana to respond to the present threat of COVID-19, particularly detecting, testing, isolating, and treating and tracing every case and contact. Others disagreed (38.6%) or expressed uncertainty (38.6%) about the ability of the health system to respond to any escalation in the disease burden. Participants highlighted the inadequacy of resources both financial, material, and human resources to contain a rise in cases and mortality. One health worker noted that *“the health system can be able (to respond to rise in the cases of COVID-19) when resourced”* (Frontline worker, interviewer 22). Those challenging the health system included inadequacy of personal protective equipment (PPE) for health professionals, limited numbers of health professionals, and limited capacity of hospitals to manage increases in cases. A majority (60.8%), however, agreed that the risk of importing new cases could be managed. Although several participants disagreed with the statement that hotspot risks had been minimized in vulnerable places, the majority believed that schools, workplaces, and other essential sites had established preventative measures.

Many respondents (47.4%) noted uncertainty as to whether communities had been fully educated, engaged, or well empowered to live under a ‘new normal’ set of conditions. Participants shared their worries that community members would not adhere to COVID-19 preventive measures without government-regulated lockdown due to low levels of knowledge about COVID-19; sociocultural barriers to observing physical distancing like handshaking, hugging, and eating with family and friends, and poor attitudes for adhering to practices including wearing masks and practicing hand hygiene. Overall, respondents’ opinions favored government mandated lockdown as an indirect reminder of the existence and extent of COVID-19, and to take precautionary measures against transmission. Most respondents (46.9%) were unsure as to whether the transmission was under control or not, and agreed that “Ghanaians in other regions were expecting their lockdown only for them to be surprised” when the government instead lifted it in the two cities. Table 3 presents the summary of the barriers and facilitators of lockdown implementation.

**T﻿able 3:** Decision-making process and barriers & facilitators of lockdown implementation

1. Policy implementation gaps • Hasty, premature lifting of lockdown driven by health system challenges, socioeconomic inequalities in access to health care and services, economic cost and impact of lockdown to the poor, the informal sector and government expenditure • Poor public health communication bolstered by inadequate evidence-based and transparency supported the decision to lift the lockdown • Poor decision to lift lockdown due to limited capacity of hospitals to manage increase in cases at that time
2. Health system barriers • Limited financial, material, and human resources to contain rise of new cases and mortality - Inadequate numbers of health workers to detect to detect, test, isolate, and treat every case and trace every contact should cases increase - Limited capacity of hospitals to manage increase in new cases - Inadequate material resources like PPEs
3. Barriers linked to sectors beyond health • Social determinants of health (intra-urban socioeconomic inequalities in access improved housing, water, toilet facilities) • Economic costs and impacts on the private informal sector • Economic costs and impacts on the government • Poor community education, engagement, and empowerment for the two city populations to live under new normal circumstances of lockdown • Sociocultural challenges to gain population’s adherence to preventive measures without government mandated restrictions
4. Facilitators • Transparency in public health communications • Adequate material, human and financial resource for health • Improved social determinants of health • Community participation and empowerment to live under new normal • Improved sociocultural practices to adhere to preventive measures even without government mandated restrictions • Evidence based decision-making • Whole-of-government approach to address barriers to lockdown implementation

## Policy implications to Ghana and the wider African context

Recent global health crises include Hantavirus Pulmonary Syndrome, Severe Acute Respiratory Syndrome (SARS), H5N1 Influenza, H1N1 Influenza, Middle East Respiratory Syndrome (MERS), and the Ebola virus outbreak [9, 10]. Preparedness, targeting disease prevention, is critical as hospitals may struggle to care for an increased magnitude of patients, especially given resource constraints. This reasoning contributed to decisions in most countries to impose restrictions due to COVID-19, including lockdowns. Even so, adequate preparation and response to outbreaks requires resources for the public health infrastructure. Hospitals and clinics have detected and treated most of those infected during COVID-19 outbreaks [11]. Based on the current WHO data, Ghana’s government expenditure on health as a percentage of total government expenditure is low, around 6.82% in 2014 [12]. COVID-19 has highlighted the often-ignored need for a massive boost in governments’ health spending, especially investment in the public health infrastructure to meet growing health needs and expectation of citizens.

An early study in Lebanon showed nationwide lockdown in 2020 had a significant impact on minimizing the spread of the pandemic and containing the virus [13]. What is an appropriate policy response or a disproportionate one? What works well in one country may not be as effective elsewhere. Lockdown implementation in some lower-middle-income countries (LMICs) proved to be effective [13], but not so in Ghana where government halted implementation due to intra-urban socioeconomic inequalities and the economic impact of lockdown on informal sector workers. Thus, we encourage policy makers to consider all contextual factors in their countries when planning a lockdown.

What might be unintended consequences on people, particularly on vulnerable groups? In many LMICs, including Ghana, lockdown led to several unplanned consequences. In two Ghanaian cities lockdown distressed those with few resources, particularly residents of the urban slums; halted activities of the informal sector, the largest sector and source of employment, and the major contributor to the national income; and slowed socioeconomic activities in major cities. Restrictions of all forms, including lockdown, bans on public gathering, religious services, schools, workplaces, and public places of entertainment, and bans on travel and sporting activities all produced socioeconomic and health adversity for people and the economy. In Ghana, these provoked swift action by the Government of Ghana to lift the lockdown.

To navigate such complexities more effectively, decision makers should move to a problem-solving strategy that addresses specific problems as part of a wider, dynamic system [14] and focuses on appropriate and sustainable solutions––an approach known as systems thinking. Systems thinking for COVID-19 will necessitate health system leaders to manage district health systems more effectively. The role of district managers is crucial: they are strategically positioned to work directly with local actors, particularly community members, to engage them in systems thinking, and strengthen health system performance and response to COVID-19.

Use of local data to generate knowledge helps policy-makers identify and effectively respond to specific challenges [15]. Information should be clearly and timely communicated to all stakeholders to foster effective, coordinated efforts against outbreaks. Communication about the core components and capabilities of public health can provide guidance needed to promote commitment for sustainable public health services, especially among the general public, academics, private sector policy-makers, regulators, public health professionals, and professional organizations [11]. Disease outbreaks, whether from an influenza epidemic or an act of bioterrorism [16], may pose unique challenges for disease detection, treatment, and prevention [17]. Often feeble decision-making capacities at the district level have contributed to poor management and coordination of health service delivery, and hindered scale-up of proven health interventions [18]. Thus, there is an urgent need to build district manager capacities for systems thinking and practice for sustainable solutions during public health emergencies.

Pandemics start with and end in the community. Community engagement is crucial for health systems preparedness and response to diseases and outbreaks. Governance in Ghana relies on both traditional and civil leaders with distinct, yet sometimes overlapping, powers and interests in managing societal problems. Although the president communicated the lockdown strategy and security agencies chiefly enforced it, traditional and civil leaders played key roles in managing transmission mitigation strategies for the COVID-19 lockdown. Communities trained and engaged in outbreak preparedness and response become vital contributors to effective detection and resilient response to disease outbreaks [16]. This includes improved adherence to prevention measures such as wearing masks, regular washing of hands, and practice of social or physical distancing–even without government-regulated restrictions.

## Conclusions

Disease outbreaks will continue to disrupt health systems and economies. Because COVID-19 lockdown in Ghana distressed the urban poor and halted activities of the informal sector, government prematurely lifted lockdown despite the increase in numbers of new cases. Even so, there is good news. Policy-makers can pursue initiatives to improve adherence to preventive measures—not just to reduce further transmission of the virus, but to sustain progress. Restrictive measures are sometimes warranted to ensure effective prevention of further spread of viruses, and to facilitate a successful detection and treatment for public safety and preservation of life. We recommend evidence-informed decision-making, effective communication and transparency, and improving preparation of country’s health systems to “detect, test, isolate, and treat every case and trace every contact.” These actions will determine how rapidly nations can succeed in the fight against COVID-19, especially in bringing economies to normality by lifting lockdowns or other restrictions.
